# Cancer-associated fibroblasts in radiotherapy: Bystanders or protagonists?

**DOI:** 10.1186/s12964-023-01093-5

**Published:** 2023-05-11

**Authors:** Inigo Martinez-Zubiaurre, Turid Hellevik

**Affiliations:** 1 Department of Clinical Medicine, Faculty of Health Sciences, UiT The Arctic University of Norway, Postbox 6050, 9037 Langnes, Tromsö, Norway; 2 Department of Radiation Oncology, University Hospital of North Norway, Postbox 100, 9038 Tromsö, Norway

## Abstract

**Background:**

The primary goal of radiotherapy (RT) is to induce cellular damage on malignant cells; however, it is becoming increasingly recognized the important role played by the tumor microenvironment (TME) in therapy outcomes. Therapeutic irradiation of tumor lesions provokes profound cellular and biological reconfigurations within the TME that ultimately may influence the fate of the therapy.

**Main content:**

Cancer-associated fibroblasts (CAFs) are known to participate in all stages of cancer progression and are increasingly acknowledged to contribute to therapy resistance. Accumulated evidence suggests that, upon radiation, fibroblasts/CAFs avoid cell death but instead enter a permanent senescent state, which in turn may influence the behavior of tumor cells and other components of the TME. Despite the proposed participation of senescent fibroblasts on tumor radioprotection, it is still incompletely understood the impact that RT has on CAFs and the ultimate role that irradiated CAFs have on therapy outcomes. Some of the current controversies may emerge from generalizing observations obtained using normal fibroblasts and CAFs, which are different cell entities that may respond differently to radiation exposure.

**Conclusion:**

In this review we present current knowledge on the field of CAFs role in radiotherapy; we discuss the potential tumorigenic functions of radiation-induced senescent fibroblasts and CAFs and we make an effort to integrate the knowledge emerging from preclinical experimentation with observations from the clinics.

Video Abstract

**Supplementary Information:**

The online version contains supplementary material available at 10.1186/s12964-023-01093-5.

## Abstract (summary)

Radiotherapy (RT) is currently used to treat more than 50% of all diagnosed cancer cases and counts for around 40% of all cure rates. The primary goal of RT has always been to induce damage on tumor cells; thus, it has been traditionally believed that the therapeutic efficacy of RT is mediated exclusively by its capacity to directly kill malignant cells. However, tumors are complex tissues composed of multiple cellular and acellular elements often referred as the tumor microenvironment (TME) or tumor stroma. Both, the malignant and the non-malignant cells in tumors receive the same prescribed radiation dose during treatment. In recent years, it has become more and more evident the fundamental role played by elements of the TME on therapy outcomes. One of the most abundant and influential elements of the reactive stroma in tumors are cancer-associated fibroblasts (CAFs). CAFs are also exposed to the full prescribed radiation dose during the course of radiotherapy. Accumulated evidence suggests that, upon radiation, fibroblasts/CAFs avoid cell death but instead enter a permanent senescent (growth arrest) state, which in turn may influence the behavior of tumor cells and other components of the TME. Despite the proposed participation of senescent fibroblasts on tumor radioprotection, it is still incompletely understood the impact that RT has on CAFs and the ultimate role that irradiated CAFs have on therapy outcomes. Further research using appropriate experimental models to study CAFs, and more information from clinical research is needed to unveil the ultimate role played by CAFs on radiotherapy.

**Supplementary Information:**

The online version contains supplementary material available at 10.1186/s12964-023-01093-5.

## Background

Radiotherapy is currently used to treat more than 50% of all diagnosed cancer cases and counts for around 40% of all cure rates [1]. Traditionally, it has been thought that the therapeutic efficacy of RT is mediated predominantly by its capacity to directly kill malignant cells. Consequently, research aiming for improved therapeutic outcomes have focused almost entirely on the cancer cell itself, encouraging dose escalation strategies or the use of radiosensitizers for enhancing the antitumor effects [1, 2]. However, in recent years, it has become increasingly evident the fundamental role played by elements of the tumor stroma on therapy outcomes [3, 4]. Hence, today, several components of the stroma have been identified to interact with the response to RT. The extracellular matrix (ECM) can dictate radiation responsiveness by influencing oxygen availability, inducing pro-survival signals via cell-matrix interactions or by controlling the bioavailability of growth factors and cytokines [5–8]. Radiation can also induce endothelial cell dysfunctions, with altered expression of cell adhesion molecules and changes in the vascular network in tumors, which altogether may translate into impaired tumor perfusion, altered immune infiltration and/or increased hypoxia [9–12]. Moreover, the inexorable tissue damage provoked by RT is normally associated with an inflammatory response [13, 14], characterized by recruitment and activation of innate and adaptive immune cells and release of potent pro-inflammatory mediators [15–17].

As one of the most abundant elements of the reactive stroma in solid tumors, cancer-associated fibroblasts (CAFs) [18–20] are exposed to the full prescribed radiation dose during the course of clinical radiotherapy [21, 22]. However, the impact that radiation has on CAFs and the potential downstream effects of radiation-induced changes on therapy outcomes remain unsettled [11, 23–25]. Studies investigating cytotoxic effects of ionizing radiation (IR) on stromal cells have revealed the intrinsic radio-resistant nature of fibroblasts [26, 27]. Several in vitro observations have confirmed that following exposure to high radiation doses, CAFs [26, 28, 29] and normal tissue fibroblasts evade cell death [30, 31], but acquire a senescent phenotype [26, 30] accompanied by impaired proliferative and migratory functions [26]. A role of senescent cells in cancer development has been proposed, however such effects can be ascribed as beneficial or detrimental depending on the context [32–38]. Based on current knowledge on the topic of radiation effects in fibroblasts, it exists a generalized view postulating the acquisition of enhanced pro-malignant functions in irradiated (senescent) fibroblasts/CAFs, irrespective of their origin and functional status [39, 40]. However, most of the existing knowledge in this field emerge from studies conducted with normal tissue fibroblasts or fibroblast cell lines. In contrast to quiescent “normal” tissue fibroblasts (NFs), CAFs (in non-irradiated conditions) actively produce numerous tumor-promoting molecules such as matrix metalloproteinases (MMPs), inflammatory cytokines, pro-angiogenic factors and miscellaneous tumor-promoting growth factors [18, 20, 41]. Thus, to understand the contribution of CAFs to therapeutic outcomes post-RT, the activated status of the cells before treatment should be taken into consideration. Additionally, in contemporary high-precision image-guided radiotherapy settings with steep dose gradients, only cells residing within the delineated tumor volume (i.e. the planning target volume), or its periphery, are exposed to the full prescribed radiation dose (i.e. CAFs) [42], whereas healthy tissues located outside the target field (NFs) normal Fbs typically receive only residual radiation doses [21, 22, 43]. In this review, we discuss the controversies and misconceptions that have emerged in this field, due to the unrecognized fact that normal tissue fibroblasts and CAFs represent two different cell entities, and that radiation effects and responses may differ substantially between them.

## Normal fibroblasts versus CAFs

Fibroblasts are spindle-shaped cells of mesenchymal origin which reside in connective tissues and are responsible for the production of ECM components and connective tissue homeostasis [44]. The embryonic origin of tissue resident fibroblasts is shared by other mesenchymal lineages including adipocytes, pericytes, chondrocytes and osteoblasts [45]. The difficulty in defining fibroblasts results largely from the lack of unique markers that are not expressed in other cell types [19, 45, 46]. In practical terms, fibroblasts are often defined by their specific cellular functions (ECM synthesis and remodeling), their lack of mutations and their lack of lineage markers for epithelial cells, endothelial cells and leukocytes. When quiescent, fibroblasts and stellate cells from specific organs do not proliferate, they form few cell-to-cell connections, they synthesize little collagen and secrete low amounts of cytokines and other signal mediators [18, 47].

Activation of tissue resident fibroblasts can occur in physiological conditions, such as wound healing or acute organ repair, or in pathologic conditions such as chronic infections, organ fibrosis, autoimmune diseases, or cancer [44, 45]. Activated fibroblasts can originate from multiple cell types including tissue-resident fibroblasts, mesenchymal progenitor cells, pericytes, organ-specific stellate cells or from trans-differentiation from different stromal cell types [18–20, 48]. Activation is triggered by multiple stimuli including hypoxia, injury-induced platelet activation and the secretion of potent factors by epithelial cells such as transforming growth factor- (TGF-β), platelet-derived growth factor (PDGF) and basic fibroblast growth factor (bFGF), resulting in increased proliferation, increased contractility, and expression of activation markers such as αSMA, PDGFRα/β, FAP, podoplanin (PDPN) and desmin [18, 20]. In the context of cancer, chronically activated fibroblasts/CAFs contribute to aberrant ECM deposition and desmoplasia, promote angiogenesis and regulate the infiltration and polarization of myeloid cells attracted to tumors [19]. A complex and intricate network of signaling pathways and crosstalk with epithelial cells and other stromal cells perpetuate the activation status of CAFs in tumors [18, 49, 50]. Most of the well-described tumor-promoting functions of CAFs happen in the context of paracrine and juxtracrine signaling via the secretion of multiple growth factors including TGF-β, connective tissue growth factor (CTGF), stromal-derived growth factor (SDF-1) vascular endothelial growth factor (VEGF), osteopontin (OPN) or hepatocyte growth factor (HGF) just to mention some, as well as a myriad of cytokines and chemokines such as IL-6, IL-8, IL-1, CXCL2, CXCL5, CXCL12/SDF-1a, CCL20 and others [18, 20, 50].

With the advent of single-cell sequencing, the complexity of CAF biology during tumor development and across different tumor entities has gradually become more evident [44, 51, 52]. CAFs, which were originally perceived as a homogenous population, are now understood to be a mixture of different fibroblast phenotypes with distinct behavior, comprising both tumor-promoting and tumor-restraining subtypes [46, 53–55]. Despite the proven existence of CAF diversity in both preclinical and clinical settings [56], the context-dependent roles of different CAF populations and their interchangeable plasticity remain largely unknown [56]. In this review, we have not put emphasis on the CAF diversity aspect since CAF heterogeneity has not been studied in the context of radiation or radiotherapy yet.

## Role of senescent fibroblasts in cancer development

Cellular senescence is an established tumor suppressive mechanism that halts the proliferation of premalignant cells [37]. Replicative senescence in a physiological context is frequently associated with aging [38, 57], and it is attributed to the gradual loss of telomere length after repeated cell divisions, whereas premature cell senescence or accelerated senescence is normally triggered by exogenous stresses, including genotoxic anticancer treatments such as chemotherapy or radiotherapy [58, 59]. Despite its tumor suppressive nature, mounting evidence indicate that senescent cells can also promote tumor progression via the senescence-associated secretory phenotype or SASP [60].

In the context of cancer, senescent cells can act as a double-edged sword. On one hand, SASP promotes tissue repair through the induction of plasticity and stemness and participates in the clearance of cancer and damaged cells by attracting phagocytes, NK cells and other immune cells [61, 62]. Induction of cell senescence in fibroblasts also limit the development of desmoplastic reactions and fibrosis [63]. On the other hand, the presence of senescent cells in the tumor stroma or its surroundings can aid in the establishment of an immunosuppressive [64, 65], pro-angiogenic [66], pro-inflammatory and catabolic microenvironment [33, 67, 68] that stimulate tumor growth and cancer cell dissemination.

Senescence-associated alterations in the secretion of matricellular proteins and ECM constituents by fibroblasts have been shown to create a favorable milieu for tumor development [33]. The observed upregulation of MMPs, cathepsins, ADAMTs (a disintegrin and metalloproteinase with thrombospondin motifs) and other proteases, together with the downregulation of tissue inhibitors of matrix metalloproteinases (TIMPs) contribute to a catabolic environment that supports tumor cell invasion and metastasis. In contrast to ECM-degrading constituents, which are mainly overexpressed in senescent fibroblasts, ECM molecules have been reported to be downregulated in general [69, 70]. Senescent fibroblasts and mesenchymal cells can also promote cancer development by favoring epithelial-to-mesenchymal transition in premalignant and malignant cells [71, 72]. This phenomenon is mediated in a paracrine fashion by the release of molecules such as MMPs, IL-1β, IL-6 and reactive oxygen species (ROS). Additionally, senescent macrophages can directly or indirectly promote tumor vascularization by overexpressed secretion of pro-angiogenic factors and by promoting the recruitment and polarization of M2-macrophages [73]. Last, the SASP from both stromal and tumor cells promotes tumor growth by establishing a microenvironment that is immunosuppressive. Such induction is mediated by the secretion of immunosuppressive (Th2) cytokines that favor the recruitment of myeloid-derived suppressor cells (MDSCs) and consequently inhibition of CD8 + T-lymphocyte-mediated killing of tumor cells [64].

## Pro-tumorigenic functions of irradiated (senescent) normal fibroblasts

Ionizing radiation has been shown to drive both stromal fibroblasts and cancer cells to premature senescence [30, 74, 75]. Accordingly, therapy-induced cell senescence has become a recognized side effect of anticancer treatments with potential to mediate substantial impact on therapy outcomes [59]. Soluble SASP elements such as MMPs have been associated with pro-tumorigenic effects from radiation-induced senescent fibroblasts in vitro and in vivo [30, 76, 77]. Moreover, insoluble (cell-associated) molecules such as syndecan-1 have also been shown to be overexpressed in radiation-induced senescent breast stromal fibroblasts, which reportedly have been demonstrated to mediate enhanced tumor progression [78].

In the context of radiotherapy, accumulated evidence reveals that on one hand, radiation-induced premature senescence instead of apoptosis is a major mode of cell fate in irradiated fibroblasts, and on the other hand, radiation-induced senescence is a dose-dependent factor, with higher doses being more effective in inducing premature senescence [26, 28]. Experiments related to the involvement of radiation-induced senescent fibroblasts in cancer promotion have usually been performed using normal human fibroblasts. In fact, the pro-malignant phenotype acquired by senescent normal fibroblasts post-RT have been thoroughly documented. Numerous in vitro studies have demonstrated increased invasiveness, proliferation rates and radio-resistance of tumor cells exposed to irradiated fibroblast cell lines (or their conditioned medium) when compared to non-irradiated cells (Table 1). Different soluble signal molecules have been proposed to be responsible for the radiation-induced enhanced effects including HGF [79], MMPs [80], TGF-β [81] as well as interleukin (IL) 6 and IL-8 [82]. There are also reports of pro-tumorigenic effects exerted by irradiated fibroblasts upon co-transplantation in in vivo models [30, 83]. Additionally, increased tumor incidence and growth have been observed in animal models when both pre-malignant and malignant cells are injected in pre-irradiated tissues [80, 84].

**Table 1:** Tumorigenic effects exerted by prematurely senescent (RT-induced) normal fibroblasts

Ref.	Fibroblast type	Tumor model	Experimental model	Effects
Rodier [82]	HCA2 NF cell line	None	In vitro (co-cultures)	Enhanced expression of IL-6 and IL-8 by IR-induced senescent fibroblasts
Ohuchida [85]	MRC5 cell line CAFs/1 donor	Pancreas	In vitro (co-cultures)	Irradiated NFs increase invasiveness of cancer cells. Increased phoshorylation of HGF receptor in tumor cells
Papadopoulou [30]	Normal human lung FBs	Lung	In vitro In vivo (subcutaneous)	Enhanced tumor growth after co-injections with IR-induced senescent fibroblasts
Kamochi [81]	NF cell lines (WI-26 VA4, NIH 3T3)	Oral SSC	In vitro	Irradiated NFs promote invasive growth of OSCC cells
Patel [79]	NFs cell lines	Esophageal cancer	In vitro	Conditioned medium from irradiated FBs promotes enhanced migration & invasion of normal epithelial cells
Tsai [80]	Primary human NF	Breast cancer	In vitro (3D-cultures)	Enhanced expression of MMPs by senescent NFs. Increased ECM catabolism
Tsai [86]	Primary human NF	Breast cancer	In vitro (co-cultures)	Increased tumor cell radio-resistance by senescent NFs
Tachiiri [27]	NFs	None	In vitro	Gene expression profiles after γ-IR
Arshad [87]	Murine lung NFs	Lung cancer	In vitro (supernatants)	Fibroblasts do NOT modulate tumor cell radio-resistance. Reduced TGF-β and MMP release after IR
Steer [88]	NF cell lines NIH-3T3, L929	None	In vitro (3D co-cultures), In vivo (co-inject)	Different outcomes with different tumor cell/fibroblasts combinations
Barcellos-Hoff [84]	Mammary gland tissue	Breast cancer	In vivo (Mammary gland irradiation)	Increased tumor growth of malignant and premalignant tumor cells in pre-irradiated mammary glands
Liu [76]	Human NFs	Suprarenal capsule	In vivo (co-injections)	Premature senescent NFs do NOT confer growth-stimulatory effects
Al-Assar [83]	PSC (non-tumoral)	Pancreatic cancer	In vitro (3D) In vivo (co-injections)	PSC enhance EMT and CSC processes via TGF-β-dependent mechanisms

Collectively, the accumulated knowledge clearly indicate that radiation of normal tissue fibroblasts may turn them pro-tumorigenic via acquisition of a senescent phenotype and the associated pro-tumorigenic SASP. Also, the accumulation of senescent fibroblasts in premalignant tissues by processes of normal aging or genotoxic stress (radiation) may create a favorable environment for tumor initiation and growth (Table 1).

## Role of (non-senescent) CAFs in radioprotection

Aiming at understanding the contribution of CAFs in tumor radio-resistance, some groups have investigated the potential radioprotective effects exerted by CAFs (non-irradiated/non-senescent) on cancer cells (Table 2). Several in vitro studies have demonstrated radioprotective effects of CAF conditioned medium on the survival and colony-forming abilities of cervical cancer [89] and pancreatic cancer cells [90]. Also, radioprotective effects from CAFs in co-culture with NSCLC cell lines have been described [91]. In preclinical models of melanoma and lung cancer, elevated expression of insulin-like growth factor 1 (IGF-1) and the chemokine CXCL12/SDF-1 by non-irradiated CAFs have been shown to be responsible for radioprotective effects on cancer cells [92]. In a study by Zhang et al. [93], CAF-derived CXCL1 was suggested to be accountable for the induction of a radioresistant phenotype in esophageal squamous cancer (ESCC), by promoting a reduction in ROS scavenging enzyme superoxide dismutase 1 (SOD-1) in cancer cells. Whereas in a pancreatic cancer model, it has been proposed that increased expression of TGF-β and possible other soluble signal molecules from pancreatic stellate cells (PSCs) promoted EMT changes in tumor cells and acquisition of a radioresistant phenotype [83]. Moreover, in a recent study by Ebbing et al. [94], using organoids and in vivo PDX models of esophageal cancer, authors point to CAF-derived IL-6 as a major soluble factor responsible for EMT induction and therapy resistance.

**Table 2:** Studies reporting on radioprotective effects exerted by CAFs (non-irradiated)

Ref.	Fibroblast type	Tumor model	Exp model	Effects
Chu [89]	CAFs (primary cultures)	Cervical cancer	In vitro co-cultures	Enhanced radio-resistance of Hela cells by conditioned medium from iCAF/tumor cell co-cultures, but not from iCAF monocultures
Hwang [90]	PSC/CAFs (immortalized)	Pancreatic cancer	In vitro supernatants	CAF-CM increase radioprotection of tumor cells. Unknown soluble factor
Wang [92]	NFs/CAFs (primary cultures)	Melanoma Lung cancer	In vitro In vivo (co-inject)	↑IGF-1, ↑CXCL12 iCAF-mediated induction of autophagy; increased ROS in tumor cells
Zhang [93]	Murine CAFs artificially induced radio-resistance	Prostate cancer	In vitro	Gene sequencing. Differentially expressed genes in radioresistant CAFs. Gene-signatures from radioresistant CAFs as predictors
Ji [91]	CAFs (primary cultures)	NSCLC	In vitro (co-cultures)	CAF-induced increased radio-resistance of tumor cell lines in vitro
Ebbing [94]	CAFs from EAC tumors	Esophageal cancer (EAC)	Human organoids Xenograft (co-inject)	Stromal IL-6 mediate therapy resistance EMT induction from CAFs. Circulating ADAM12-levels correlate with IL-6 expression by CAFs and bad prognosis

In clinical settings, the enhanced expression of CAF-specific markers or CAF-related gene signatures in tumor specimens have shown potential to predict responses to radio(chemo)therapy, and is persistently associated with poor prognosis in different types of cancers, including prostate cancer [95], cervical cancer [96], breast cancer [97], colon cancer [98], oral squamous cell carcinoma [99] and rectal cancer [100] (Table 3). Such consistency in clinical findings suggest that CAFs may play important roles in the conferral of radiation resistance phenotypes across many different solid malignancies.

**Table 3:** Studies reporting on CAF-related predictive/prognostic markers in clinical radio(chemo) therapy

Ref.	Tumor type	Fibroblast marker	Experimental model	Clinical relevance
Zhang [95]	Prostate cancer	Gene signature	Transcriptomics	Radioresistant CAF signature predict worse outcomes
Kim [96]	Cervical cancer	Gene signatures	Transcriptomics	CAF gene-signature associated with poor prognosis & worse outcomes
Strell [97]	Breast Cancer (DCIS)	PDGFR-β	TMA IHC	PDGFR-β stroma expression is predictive of bad RT outcomes
Verset [98]	Colo-rectal cancer	α-SMA Ki-67	IHC pre-& post-op	Enhanced α-SMA/epithelial ratio after RT. ↑ α-SMA /epithelial ratios correlate with bad clinical outcomes
Matsuoka [99]	Oral squamous cell carcinoma (OSCC)	α-SMA CD163	IHC whole specimens	High abundance of CAFs & TAMs correlates with poor prognosis and bad response to neoadjuvant CRT
Saigusa, [100]	Rectal cancer	FAPα SDF-1	Gene expression pre-op samples	Enhanced expression of FAPα and SDF-1 correlates with poor prognosis

## Effects of RT on CAF tumorigenic functions

In addition to the general radioprotective functions assigned to CAFs, some studies claim that radiation exposure is amplifying the intrinsic radioprotective and pro-malignant effects exerted by CAFs (Table 4). In a study by Li et al. [101], irradiated CAFs provoked induction of epithelial mesenchymal transition (EMT) and enhanced invasive capacity of pancreatic cancer cells in co-cultures. RT-activated CAFs were found to excrete increased levels of CXCL12/SDF-1, ultimately promoting a mesenchymal phenotype in cancer cells and aiding to the overall tumor progression. Again, in a pancreatic cancer model, Mantoni et al. [102] demonstrated that pancreatic stellate cells (PSC) promote radioprotection and stimulate proliferation of pancreatic cancer cells in direct co-cultures and after co-injections in animal models. In this study, interfering with β1-integrin signaling abolished the radioprotective effects. In a third preclinical study performed on pancreatic cancer, authors show that induction of iNOS and nitric oxide (NO) in CAFs by RT is responsible for elevation of intratumoral pH and induction of a proinflammatory phenotype in tumor cells in a NFκB-dependent manner [103]. In a colorectal cancer model, Tommelein et al. [104] found increased IGF signaling from irradiated CAFs, and both IGF1 and IGF-binding proteins (IGFBP2) levels were elevated in supernatants from irradiated versus non-irradiated CAFs. In another preclinical study of colorectal cancer (CRC), upon irradiation, tumor-derived interleukin1α (IL-1α) mediated polarization of cancer-associated fibroblasts (CAFs) towards a pro-inflammatory pro-tumorigenic phenotype [105]. Authors demonstrated that IL-1-dependent signaling elevates oxidative DNA damage in iCAFs, which upon irradiation undergo senescence. This causes tissue remodeling and therapy resistance that can be overcome by inhibiting IL-1. Finally, in a recent study by Meng et al. [106], authors demonstrated that radiation-induced senescent CAFs promote non-small cell lung cancer (NSCLC) cell proliferation and radio-resistance through activation of the JAK/STAT pathway in tumor cells. Selective targeting of senescent fibroblast with a senotoxic agent was able to radio-sensitize tumors.

**Table 4:** Studies reporting on pro-& anti-tumorigenic effects exerted by irradiated CAFs

Ref.	Tumor type	Fibroblast type	Experimental Model	Effects
Li [101]	Pancreatic cancer	CAFs & NFs	In vitro (co-cultures) In vivo (co-injections)	iCAFs enhance tumor cell invasion and promote EMT. ↑SFD-1
Mantoni [102]	Pancreatic cancer	PSC LTC-14	In vitro (co-cultures) In vivo (co-injections)	CAF-mediated increased radioprotection of tumor cells. Integrin-β signaling
Pereira [103]	Pancreatic cancer	CAFs primary cultures	Orthotopic implantation of clinical specimens	iNOS/NO expression from iCAFs increase intra-tumoral pH and tumor growth. Activation of NF-kB and secretion of cytokines in tumor cells by CAF-CM
Tommelein [104]	Colo-rectal cancer	CAFs	In vitro (supernatants) In vivo (co-injections)	Increased IGF-1 secretion; iCAFs promote cancer cell survival and radioprotection
Nicolas [105]	Rectal cancer	CAFs in organoids	In vitro (PD organoids)	Radiation-induced IL-1a in tumor cells provoke polarization of CAFs towards inflammatory/pro-tumorigenic phenotype
Meng [106]	Lung cancer	CAFs primary cultures	In vitro co-cultures In vivo (co-injections)	Pro-tumorigenic effects from IR-induced senescent CAFs. Pharmacological targeting of senescent-like CAFs radio-sensitize tumors
Grinde [107]	NSCLC	CAFs primary cultures	In vivo co-injections	Reduced pro-tumorigenic functions by irradiated CAFs in vivo
Hellevik [108]	NSCLC	CAFs primary cultures	In vitro co-cultures	Irradiated CAFs do NOT enhance proliferative and migratory functions on tumor cells

Conversely to the current view proposing a radiation-enhanced activation of CAFs, some studies document a loss of CAF pro-tumorigenic functions after irradiation (Table 4). In an in vivo study by Grinde et al. [107], the tumor enhancing effects exerted by NSCLC-CAFs after co-injection with A549 cells in nude mice was lost when CAFs were irradiated prior to implantation. In an in vitro study performed with NSCLC-CAFs, authors demonstrate changes in the secretory profile CAFs upon a single-high dose (1 × 18 Gy) radiation. However, in functional assays, they report no effects of conditioned media from irradiated CAFs on the proliferative or migratory capacity of tumor cells, and reduced migration rates on endothelial cells (HUVECs) [108]. Arshad et al. [87] reported similar findings, showing that murine lung CAFs did not affect the intrinsic radio-sensitivity of cancer cells. In contrast, reduced expression of TGF-β and MMPs were observed in co-culture supernatants after exposure to (1 × 10 Gy) radiation. In another study by Steer et al., the radio-protective and long-term survival effects of CAFs over cancer cells were studied in 2D and 3D in vitro systems, using different sets of fibroblasts and tumor cell lines [88]. The outcomes were inconsistent among different fibroblast-tumor cell combinations. Similar observations were obtained after co-implantation of cells in xenografts. Some authors have suggested that cancer promotion by senescent stromal cells may be restricted to certain organs and tissue types and claim that the tumorigenic properties of senescent cells need to be validated in other tissues than subcutaneous lesions [109].

## CAFs impact on RT and vice-versa: observations from the clinics

In clinical settings, radiotherapy continue being a safe and efficient way to treat most known solid malignancies [1]. Exacerbated tumor growth after curative, adjuvant or neoadjuvant (chemo)radiotherapy is scantly observed, even though local tumor progression under or post-treatment may occur in minor subgroups of patients. In contrast, improved outcomes are frequently observed when radiation is used pre- or post-operative as opposed to surgery alone. Such long-term benefit from RT treatment have been reported for many different types of malignancies, ranging from early-stage breast cancer [110] to locally advanced colorectal cancer [111]. Considering that nearly all solid neoplasms contain tumor stroma and CAFs at all developmental stages, the evidence from the clinics suggests that the potential activation of CAFs post-RT (if happening at all) is not a dominant force that dictate the fate of the therapy. On the other hand, there is not much evidence on the impact of radiotherapy on CAFs in clinical settings. A study by Verset et al. on rectal cancer explored the impact of (chemo)radiotherapy on CAFs by comparing α-SMA/KI-67 ratios in tumor specimens collected before and after radiotherapy. Results showed that the α-SMA/neoplastic epithelial area ratio was higher after neoadjuvant therapy, and that α-SMA/epithelial area ratio was an adverse prognostic factor regarding recurrence-free survival [98]. Studies showing prognostic and predictive potential of CAF-markers in the clinics have been presented in the previous chapter (Table 3).

## Concluding remarks

Scientific efforts over the last couple of decades have uncovered the important role played by the different elements of the tumor stroma in (radio)therapy outcomes. Cancer-associated fibroblasts have been proposed to participate significantly in tumor responses to radiotherapy. However, despite their prominent participation in tumor development and therapy resistance, the impact of RT on CAFs and the role of CAFs on RT outcomes remain elusive. Most existing literature in this field argue that radiation mediate changes in the mesenchymal components that favor tumor establishment and progression, however the impact that RT has on CAFs in vivo and the downstream effects of the potential RT-mediated changes remain controversial and still insufficiently investigated.

A potential source of controversy may emerge from the unrecognized fact that CAFs and normal fibroblasts are different cell entities, behaving differently, and probably responding differently to radiation exposure even when considering that both may survive and turn senescent after exposure to substantial radiation doses. Collectively, most published studies on the effects of radiation performed with normal tissue fibroblasts or fibroblast cell lines demonstrate enhanced pro-tumorigenic functions from irradiated (senescent) cells induced primarily in a paracrine fashion, by increased secretion of soluble growth factors, inflammatory mediators, and proteolytic enzymes (Table 1). Moreover, numerous studies have documented enhanced radioprotection of tumor cells cultured in the presence of (non-irradiated) CAFs (Table 2). These observations are in accordance with other studies performed on clinical specimens, demonstrating clear associations between high expression levels of CAF markers or CAF signature genes and poor prognosis (Table 3).

Findings on the impact of RT on CAFs and on the role of irradiated CAFs on tumor cell behavior are more controversial. While some studies claim that radiation exposure may affect CAF negatively through growth arrest and impaired mobility, others argue that exposure of CAFs to radiation can promote a more aggressive phenotype capable of conferring enhanced radio-resistance on tumor cells (Table 4). Most published studies have been performed on in vitro culture conditions, using single radiation doses or regimens, and have collected data at specific time-points, normally few hours/days post IR. These approaches gloss over potential differences related to different RT-regimens, that are crucial factors in modern radiotherapy [112] and radiotherapy-immunotherapy settings [4, 113–116] and also overlook potential long-term effects of RT on CAFs. Besides, most preclinical in vivo studies use co-injections of tumor cells and fibroblasts/CAFs orthotopically or in subcutaneous pockets. In transplantation experiments with admixed cells, it is frequently observed that non-tumoral cells disappear quickly after implantation [76, 107, 117]. Tumor growth effects in such experiments are believed to be related to initial tumor engraftment rather than tumor growth, and are therefore not optimal to study effects from transplanted (irradiated) cells. Additionally, there is little or no evidence on the impact that RT has on CAFs in vivo (preclinical) or in clinical settings. Preclinical models reproducing stroma-rich tumors resembling the human scenario, where endogenous CAFs can be targeted, tracked and/or regulated, could represent attractive models to explore CAF-mediated effects from RT and vice versa. In future efforts, the CAF heterogeneity aspect, including aged-fibroblasts, should also be taken into consideration. It is still unknown which CAF subtype (if any) is mostly responsible for conferring tumor cell radio-resistance. We still don’t know if radiation affects all CAF subtypes in the same way, or if the induction of cell senescence by RT unifies all subpopulations into a single CAF cell phenotype with specific functions.

In the clinics, radiotherapy is considered to be a safe and effective way to treat non-metastatic cancers with curative intent. Moreover, the use of RT in adjuvant and neoadjuvant settings has been proved to give better survival rates than surgery alone in many different tumor types [1]. Considering that basically all solid neoplasms contain CAFs at all developmental stages, the evidence from the clinics suggests that the potential activation of CAFs post-RT is not a dominant force that dictate the fate of the therapy. At least, it remains unexplained why local tumor recurrence or exacerbated tumor growth is not more frequently observed following RT, if we assume that radiation triggers enhanced pro-tumorigenic functions in CAFs. Further research using appropriate models to study CAFs and more information from clinical research is needed to clarify the ultimate role played by CAFs on radiotherapy.

## Search strategy and selection criteria

Data for this review were identified by searches of MEDLINE, PubMed and references from relevant articles using search terms such as “cancer-associated fibroblasts”, “tumor microenvironment”, “radiation”, “radiotherapy”, “cell senescence”, “stress-induced senescence”. Only articles published between 1999 and 2022 have been included.

## Data Availability

Data sharing not applicable to this article as no datasets have been generated or analyzed for the creation of the study.

## Abbreviations

α-SMA: Alpha-smooth muscle actin
CAFs: Cancer-associated fibroblasts
ECM: Extracellular matrix
EMT: Epithelial-mesenchymal transition
IR: Ionizing radiation
MMPs: Matrix metalloproteinases
NFs: Normal fibroblasts
NSCLC: Non-small cell lung cancer
RT: Radiotherapy
SASP: Senescence-associated secretory phenotype
TME: Tumor microenvironment
